# A deep learning approach for facility patient attendance prediction based on medical booking data

**DOI:** 10.1038/s41598-020-71613-7

**Published:** 2020-09-03

**Authors:** Francesco Piccialli, Salvatore Cuomo, Danilo Crisci, Edoardo Prezioso, Gang Mei

**Affiliations:** 1 Department of Mathematics and Applications “R. Caccioppoli”, University of Naples Federico II, 80126 Naples, Italy; 2 School of Engineering and Technology, China University of Geosciences (Beijing), Beijing, 100083 China

**Keywords:** Health services, Mathematics and computing

## Abstract

Nowadays, data-driven methodologies based on the clinical history of patients represent a promising research field in which personalized and intelligent healthcare systems can be opportunely designed and developed. In this perspective, Machine Learning (ML) algorithms can be efficiently adopted to deploy *smart* services to enhance the overall quality of healthcare systems. In this work, starting from an in-depth analysis of a data set composed of millions of medical booking records collected from the public healthcare organization in the region of Campania, Italy, we have developed a predictive model to extract useful knowledge on patients, medical staff, and related healthcare structures. In more detail, the main contribution is to suggest a Deep Learning (DL) methodology able to predict the access of a patient in one or more medical facilities of a fixed set in the immediate future, the subsequent 2 months. A structured Temporal Convolutional Neural Network (TCNN) is designed to extract temporal patterns from the administrative medical history of a patient. The experiment shows the goodness of the designed methodology. Finally, this work represents a novel application of a TCNN model to a multi-label classification problem not linked to text categorization or image recognition.

## Introduction

Healthcare is one of the main sectors fostering the exponential growth of big data on account of four important phenomena: the digitalization of diagnostic imaging, the replacement of papers with digital reporting, the development of biotechnologies used in the field of the so-called “omics” sciences, and the explosion of the so-called Internet of Medical Things (IoMT). The application of Machine Learning (ML) in healthcare is a very promising research field in which researchers, companies, and organizations are increasingly endeavoring to design innovative services and smart solutions^1,2^. The potential of ML in medicine is particularly notable in certain areas, such as the automatic analysis of medical records, which, being compiled in an unstructured way, have traditionally not been considered as exploitable with an algorithmic approach aiming at the production of automatic and structured composition reports. The progress of ML, on the other hand, is making it possible to exploit these data also, since it is no longer as “difficult” for software as it had been until the recent past. In this perspective, the great experience and huge amount of data deriving from the healthcare domain can enable physicians and organizations to make quicker and more accurate diagnoses and offer personalized and efficient services^3^ and can facilitate researchers in an understanding of the mechanisms underlying diseases to predict the disease risk and achieve its timely prevention. Yang et al. discussed for the first time on the application of emerging information technologies and new paradigms to healthcare services^4^. Certainly, a potentially immense amount of data is generated, enhanced day-by-day through the application of e-Health services, such as Electronic Health Records (EHRs), and the storage of the medical appointments, diagnoses, and prescriptions of each patient, managed through medical management software used by healthcare facilities and health professionals. Designing predictive models using EHRs^5,6^ is also a well assessed research direction. Here the main issue is the extraction of predictive variables from the available data in each medical patient’s record. In a data-driven healthcare regime, many challenges have to be addressed, such as temporality, sparsity, noisiness, and bias on the EHR data. Accordingly, several ML approaches based on deep learning methodologies have been proposed^7,8^. Since 2012, the accesses to medical services within the public healthcare system in Italy are provided through a booking system in dedicated centers administered by the local health authority and controlled by the regional government. To access the booking center, a referral written by a practitioner is required; each referral includes a prescription to different provisions, like specialized medical examinations, medical therapy sessions, laboratory testing analyses, e.g. any venous blood sampling examinations, and diagnostic examinations. The data that we have analyzed in this research study comes from a distributed database serving various local health departments of Campania, Italy. In more detail, we have analyzed data generated within five years, from 1st January 2014 to 31st December 2018, of medical prescriptions and booking appointments, including cancellations and reschedulings, which in total amount to more than 13 million entries. This paper aims to exploit temporal administrative records to provide predictions on the possible medical examinations of a patient in the following two months, and in particular at which facility the appointment will occur. Thus, the model is linked to the prediction of patient distribution through the regional healthcare system. Therefore, our problem is a *multi-label problem*, because an appointment booking at one facility does not exclude the possibility of another appointment at a different facility. In this work, the number of facilities we focused on is fixed to 10. Our ML approach consists of a deep learning methodology based on a Temporal Convolutional Neural Network (TCNN), adapted to the more complex case of patient information developing over time. The output of the proposed method is, for each facility under consideration, the possibility that the patient will have an appointment at that facility, expressed as a percentage. The performance of our model has been verified by considering several metrics in a comparison with other methodologies suited to the solution of multi-label problems. Moreover, in the latter part of the work, an additional service is presented: a lower bound for each facility patient attendance in the subsequent 2 months.

## Data preprocessing

The main table of the database has more than 13 million unique rows, referring to booking appointments and medical prescriptions covering the period 2014–2018. Each record stores insights about the patient (gender, age at the time of prescription, etc), the practitioner, the appointment [date, medical facility, health service (HS) provision, etc], and the referral (prescription date, number of prescriptions, etc). Moreover, code correspondence tables contain details about each medical facility (e.g. location) or HS provision (e.g. a list of medical branches that can be associated together).

### Data cleaning

Due to the presence of outliers in the data set (e.g. test referrals with more than one patient and/or multiple practitioners), an operation of data cleaning has been carried out: (1) all records containing ‘unknown’ referrals, prescriptions, locations and medical facilities (due to unrecoverable errors in the encoding of the identification string) were removed; (2) only appointments labeled ’Valid’ were maintained; (3) only patients with at least five appointments throughout the reference period were maintained; (4) all invalid records containing zero appointments were removed; and (5) all records having negative days of waiting between the date of registration and the date of appointment were removed. This operation reduced the number of records from around 13 million to around 8.4 million and the number of patients from around 1.6 million to around 500,000.

### Data analysis

Firstly, we analyzed the number of unique occurrences of each interested entity.

**Table 1:** The table records the totals of unique occurrences of each entity of interest, constructed in order to obtain a first insight of the data set.

Entity	Number of unique occurrences
Appointments	8,426,972
Patients	491,137
Health service provisions	2,382
Appointment dates	1,720
Medical facilities	369
Medical branches	28

For example, there are more than 2,000 different HS provisions and nearly 400 distinct medical facilities.

**Figure 1:**
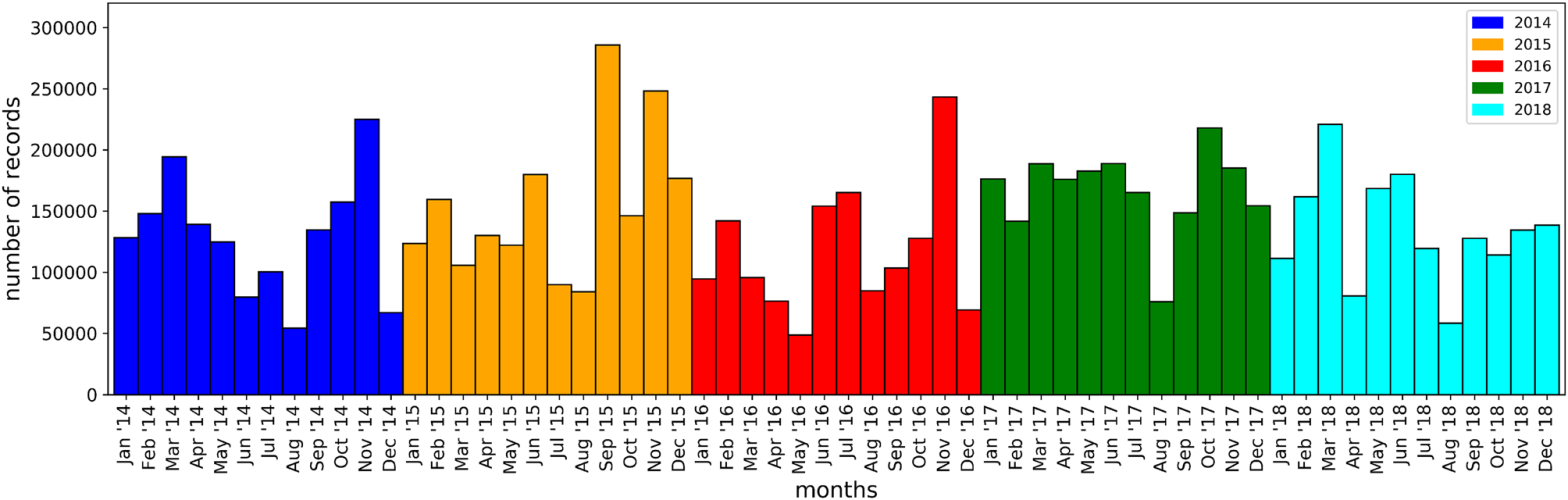
The histogram shows the number of records relating to each month of the examined period, 2014–2018. The month with the minimum value, May 2016, contains, however, a significant number of records, nearly 50,000 appointments.

As can be observed from Table 1, the data set is characterized by a *high heterogeneity*. Therefore, we investigated the frequencies to obtain a better insight into the entity distribution. Regarding the appointment dates, the histogram in Fig. 1 shows that there is a significant number of appointments in any considered year or month, a fact which led us not to be concentrated only on the data of the previous years.

Concerning the HS provisions Table 2a records the first six, ordered by frequency. From Table 2b it can be observed that more than 70% of the HS provisions (1,815 out of 2,521) have an overall frequency lower than 1%. However, it is also clear that considering only one hundred results in a significant information loss. Thus, our data set is also characterized by *sparsity*.

**Table 2:** Frequencies of health service provisions.

Type of provision	Frequency
**(a)**	
Diabetes follow-up	0.0449
ECG	0.0406
Orthopedic examination	0.0281
Cardiac examination	0.0231
Venous blood sampling	0.0219
Dilated fundus examination	0.02

Top n provision	Cumulative frequency
**(b)**	
50	0.592
100	0.753
150	0.841
200	0.889
344	0.95
706	0.99

Table (a) shows the HS provisions with the highest frequencies; Table (b) shows the cumulative frequency of the top n provisions with different values of n. It can be observed that considering only a few hundred HS provisions would imply a loss of substantial information.

### Data manipulation

After the data analysis, the following features were extrapolated for each patient: gender, birth year (estimated by subtracting the year of the prescription date and the patient age for each record and taking the minimum value), and an appointment list in terms of HS provision and medical facility for each date. To incorporate temporal information, inspired by the work of Cheng et al.^8^, we chose a matrix representation with regular time intervals; since more than 97% of the patients never had more than two distinct appointments in the same week, we selected the weeks as time steps (number of weeks: 262). For each patient, two matrices in a sparse form, named $\mathtt {P}$ and $\mathtt {F}$, were generated, with the time step as the row index, with the HS provision id and medical facility id as the column index, respectively. Therefore, for each patient, we built two matrices $\mathtt {P} \in {\mathbb {N}}_0^{262 \times 2382}$ and $\mathtt {F} \in {\mathbb {N}}_0^{262 \times 369}$, where for example $\mathtt {P}_{88,54}$ is the number of appointments in the 88th week of the period 2014–2018 relating to the 54th HS provisions, $\mathtt {F}_{133,207}$ is the total of appointments in the 133rd week booked in the 207th medical facility. Since our interest was to build a model which considers only the previous twelve months of the patient’s medical history, we decided to adopt the following approach to exploit the entire data set as effectively as possible: for each patient, any possible 52-week temporal window of his medical history (1st–52nd week, 2nd–53rd week, etc) was extracted; only if there were appointments in at least four distinct weeks of this period and if there was at least one appointment in the following 2 months (9 weeks), was the window kept and the sub-matrices of $\mathtt {P}$ and $\mathtt {F}$ relating to the same period stored. Holding the same notation, we have $\mathtt {P} \in {\mathbb {N}}_0^{52 \times 2382}$, $\mathtt {F} \in {\mathbb {N}}_0^{52 \times 369}$ and $\widetilde{\mathtt {F}} \in {\mathbb {N}}_0^{9 \times 369}$ (relating to the following 2 months, used to create the labels) for each window. Due to the high dimensionality and sparsity of the data, we made the following considerations. At first, we gathered the corresponding medical branches of each HS provision into a few groups. However, unfortunately, we checked that an HS provision can be associated with different branches, and therefore this kind of collection does not result in a partition of the HS provisions set. Therefore, we constructed some statistical features of the HS provisions to cluster them into eight clusters (the number of clusters was determined by using nbClust routine^9^, the clustering was performed by using K-means). Regarding the medical facilities, we sorted them by the amount of the appointments collected in the $\widetilde{\mathtt {F}}$ matrices; due to sparsity, this step aims to classify samples with not extremely unbalanced labels. We considered the top 10 medical facilities as the targets for the multi-label classification problem; Table 3 illustrates them.

**Table 3:** The top 10 medical facilities in Naples, Campania, sorted by the number of appointments in the extracted windows during the preprocessing phase, from 2014 to 2018.

	Medical facility	Number of appointments
1	PSI Napoli Est	399448
2	PSP C.so Vittorio Emanuele	354489
3	PSP Santa Maria di Loreto Crispi	349398
4	Poliambulatorio Scampia	311289
5	Poliambulatorio Palazzo Ex INAM	301247
6	Presidio San Gennaro	298928
7	Poliambulatorio Winspeare	289084
8	Poliambulatorio Cesare Battisti	274538
9	Ambulatorio San Gennaro ad Antignano	261734
10	PO Dei Pellegrini	248924

We also used these ten facilities to reclassify the remaining 359 ones according to their location. In detail, the top 10 facilities are located in eight distinct districts; therefore, the others were rearranged into nine groups basing on their geographical position (eight groups which gather the medical facilities of the same district and a “rest-of-the-universe” group). Thus, the newly generated data set had about 7.1 million records, each one consisting of seven features: (1) patient id, (2) window progressive id, (3) gender, (4) age at the first week of the window, (5) matrix $P \in {\mathbb {N}}_0^{52 \times 8}$ (HS provision cluster id as column index), (6) matrix $F \in {\mathbb {N}}_0^{52 \times 19}$ (an integer from 1 to 10 + 8 + 1 as column index, representing top 10 ranked facilities, the groups of facilities for each of the eight districts and the “rest-of-the-universe” group), (7) matrix ${\widetilde{F}} \in {\mathbb {N}}_0^{9 \times 19}$. For example, $P_{63,4}$ is the number of appointments in the 63rd week of the period 2014–2018 relating to the HS provisions of the fourth cluster, $F_{126,7}$ is the total of appointments in the 126th week booked in the seventh medical facility, and $F_{174,15}$ is the number of appointments in the 174th week booked in a facility of the fifth district.

**Figure 2:**
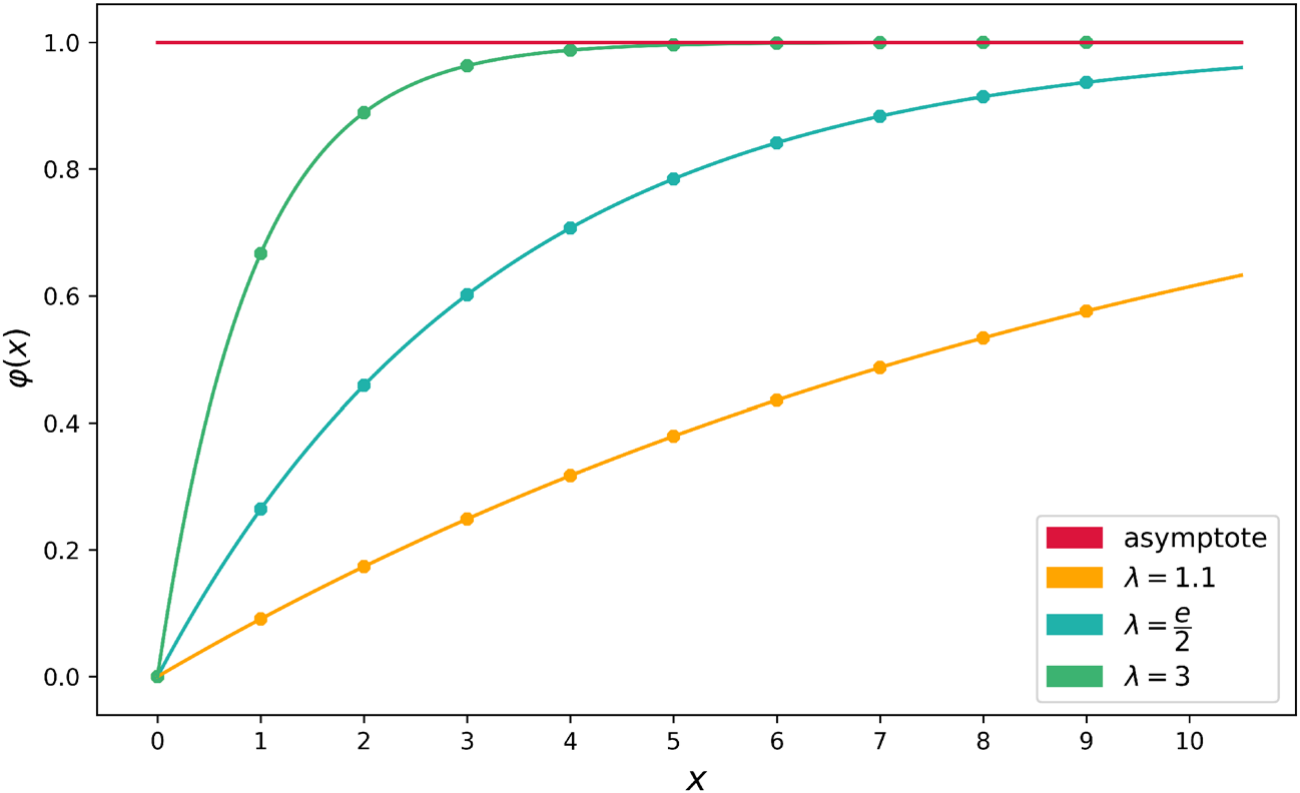
Plot of the function $\varphi (x) = 1 - \lambda ^{-x}$ with various values of $\lambda$, including, for illustrative purposes only, the delimiting line at $y = 1$. Dots have been added to aid the visualization of the mapped values of the integers. As parameter $\lambda$ increases, the distance of $\varphi (0)$ and $\varphi (1)$ increases while the difference between the mapped values of the higher integers decreases.

Next, since the features have different ranges, a scaling operation of data into the interval [0, 1] was required: gender feature ($0 = \hbox {male}$, $1 = \hbox {female}$) without any need for further mapping; age was scaled with a min–max normalization ($\hbox {min} = 0$, $\hbox {max} = 120$); and for the *P* and *F* elements scaling, we introduced a non-linear parametric scaling $\varphi$ to emphasize the differences between the low numbers rather than between the high ones. In particular, the map defined was $\varphi : x \in {\mathbb {N}} \mapsto \varphi (x) = 1 - \lambda ^{-x} \in [0,1[$ with $\lambda = \frac{e}{2}$; the Fig. 2 shows how the graph of $\varphi$ depends on the parameter while the Table 4 illustrates the mapped values of the first integers for the chosen value of $\lambda$.

**Table 4:** The table records the values of the function $\varphi (n) = 1 - \lambda ^{-n}$ with $\lambda = \frac{e}{2}$ and the nearest backward finite difference in order to highlight how the distribution and the distance of the mapped points change.

*n*	$\varphi (n)$	$\varphi (n) - \varphi (n-1)$
0	0	–
1	0.264241	0.264241
2	0.458658	0.194417
3	0.601703	0.143044
4	0.706949	0.105246
5	0.784385	0.077436
6	0.841359	0.056974
7	0.883279	0.041919
8	0.914121	0.030842
9	0.936814	0.022693

### Data labeling

Based on ${\widetilde{F}}$, each window was labeled with a vector $y \in \{0,1\}^{11}$, as follows:

$$\begin{aligned} y_j = \left\{ \begin{array}{ll} 0 &{} \text {if }\quad {\widetilde{F}}_{i,j} = 0 \; \text { for each } i=1,\dots , 9 \\ 1 &{} \text {if } \quad \exists \, i \in \{1,\dots ,9\} : {\widetilde{F}}_{i,j} > 0 \\ \end{array} \right. , \quad \text { for } j=1,\dots , 10 \end{aligned}$$

while $$y_{11} = 0$$ only if $${\widetilde{F}}_{i,j} = 0$$$$\forall i \in \{1,\dots ,9\}$$$$\forall j \in \{11,\dots ,19\}$$; otherwise $$y_{11} = 1$$. According to this definition, for $$j \in \{1,\dots ,10\}$$ the *j*-th label is equal to 1 if the patient had at least one appointment in the *j*-th facility in the following nine week period; otherwise, the label is 0. The 11th label was added only because the proposed method needs to handle non-zero label vectors.

In conclusion, the final data set handled by the classification methods is summarized in Tables 5 and 6.

**Table 5:** The input columns of the provided final data set for multi-label classification.

Feature	Type	Description
gender	int	Patient gender. 0 if male, 1 if female
age	double	Patient age
$\mathtt {P}$	int [$\mathtt {52 \times 8}$]	Patient provision history
$\mathtt {F}$	int [$\mathtt {52 \times 19}$]	Patient facilities history

**Table 6:** The output columns of the provided final data set for multi-label classification.

Label	Type	Description
$y_1,\dots ,y_{10}$	int [$\mathtt {10\times 1}$]	$y_j = 1$ if the patient makes at least an appointment in the facility *j* in the next two months, 0 otherwise
$y_{11}$	int	1 if the patient makes at least an appointment in any other facility than the top 10, 0 otherwise

## Methods and metrics

This study aims to provide predictions on the possible appointments of a patient at any considered facility in the subsequent 2 months, starting from the patient’s clinical history from the previous year. In particular, to validate the proposed predictive model, we made a comparison with some traditional methods, with particular attention to the multi-label nature of the problem. In any case, where a *Neural Network* is deployed, the adopted back-propagation strategy is based on the *BP-MLL* method^10^. It is important to note that such a method can only work when there are no samples with an output of all zeroes or all ones, and for this reason, there are 11 labels, where the last one represents the “other” facilities, i.e. the complement of the first 10 labels representing the top 10 facilities. The adopted threshold function for each label after the training is the same as in equation 20 of the aforementioned work, which minimizes the sum of false positives and false negatives in the validation set.

### Traditional methods

The two temporal windows are represented as two vectors, which are the flattened versions of the temporal window matrices. These two vectors, concatenated together with the patient’s features, which are gender and age, represent the input for the following methods: *Random Forests* (RF) and *Multi-Layer Perceptron Neural Network*, (MLP). RF^11^ is a bagging algorithm^12^ which produces multiple random predictors based on decision trees^13,14^, while MLP is a collection of *perceptron* neurons^15,16^ arranged in dense layers and linked in a suitable way, in order to capture impulse responses by adopting a feedforward approach^17^. It is important to note that other Neural Networks, like the ones based on the *Recurrent Neural Networks*, were not used, due to the known fact that their internal memory mechanisms do work very well only on consequential data without large “holes”, i.e. there are not large portions of the input filled with zeroes. Furthermore, we did not use other known traditional Machine Learning methodologies like *Support Vector Machines* (*SVM*) and *K-Nearest Neighbors* (*KNN*) because they are not naturally suited to multi-label classification problems without having to use strategies like one-vs-one or one-vs-all.

### Proposed method

**Figure 3:**
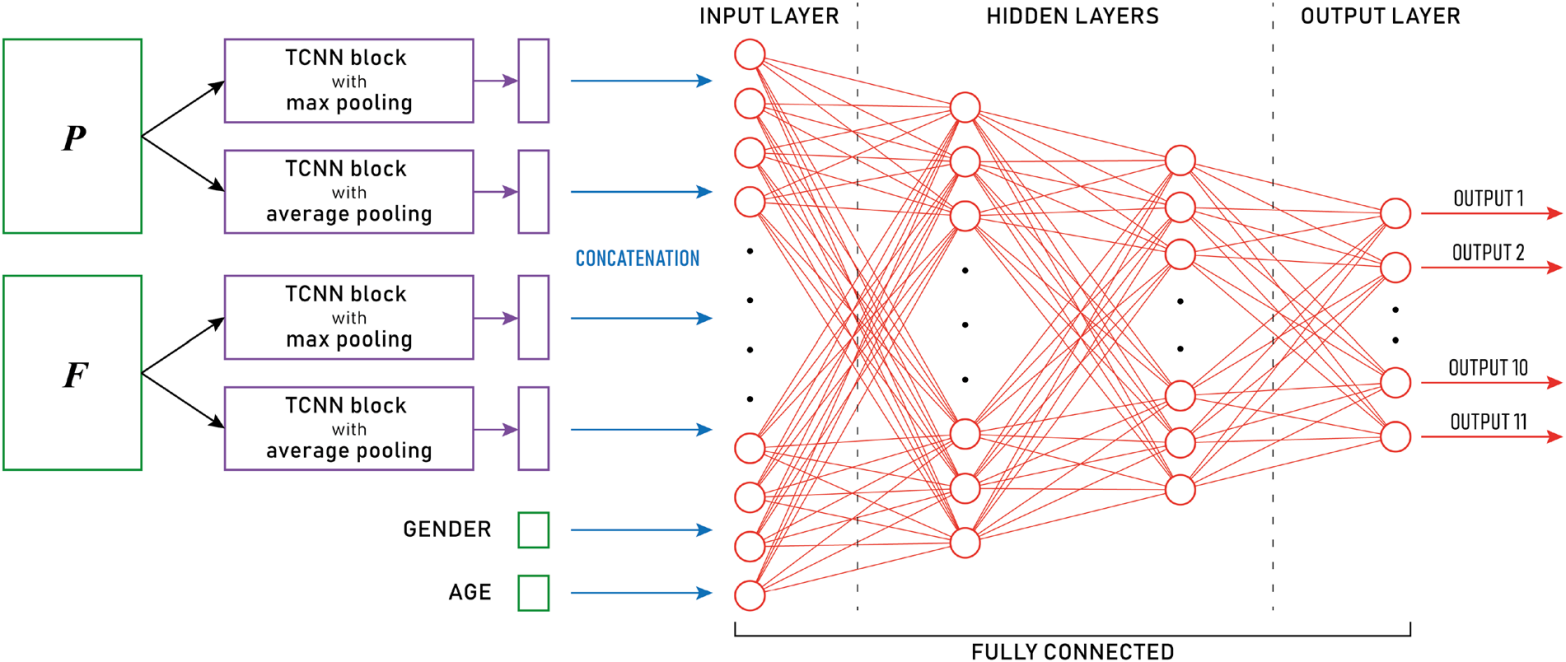
The proposed network model. The boxes in green represent the blocks of information in the input, i.e. $P$, $F$, gender, and age, where $P$ is a temporal matrix referring to the HS provisions and $F$ is a temporal matrix referring to the facilities. Gender and age are placed away from $P$ and $F$ to highlight the need to first extract hidden patterns from $P$ and $F$. In detail, from the left side: $P$ and $F$ are each passed through two parallel TCNN blocks, where each block is characterized by its pooling layer. The outputs of the four blocks are concatenated together with gender and age and passed through a fully connected neural network with two hidden layers. Each circle represents a dense neuron and the links provide the feedforward propagation from the previous neuron to the next neuron.

**Figure 4:**
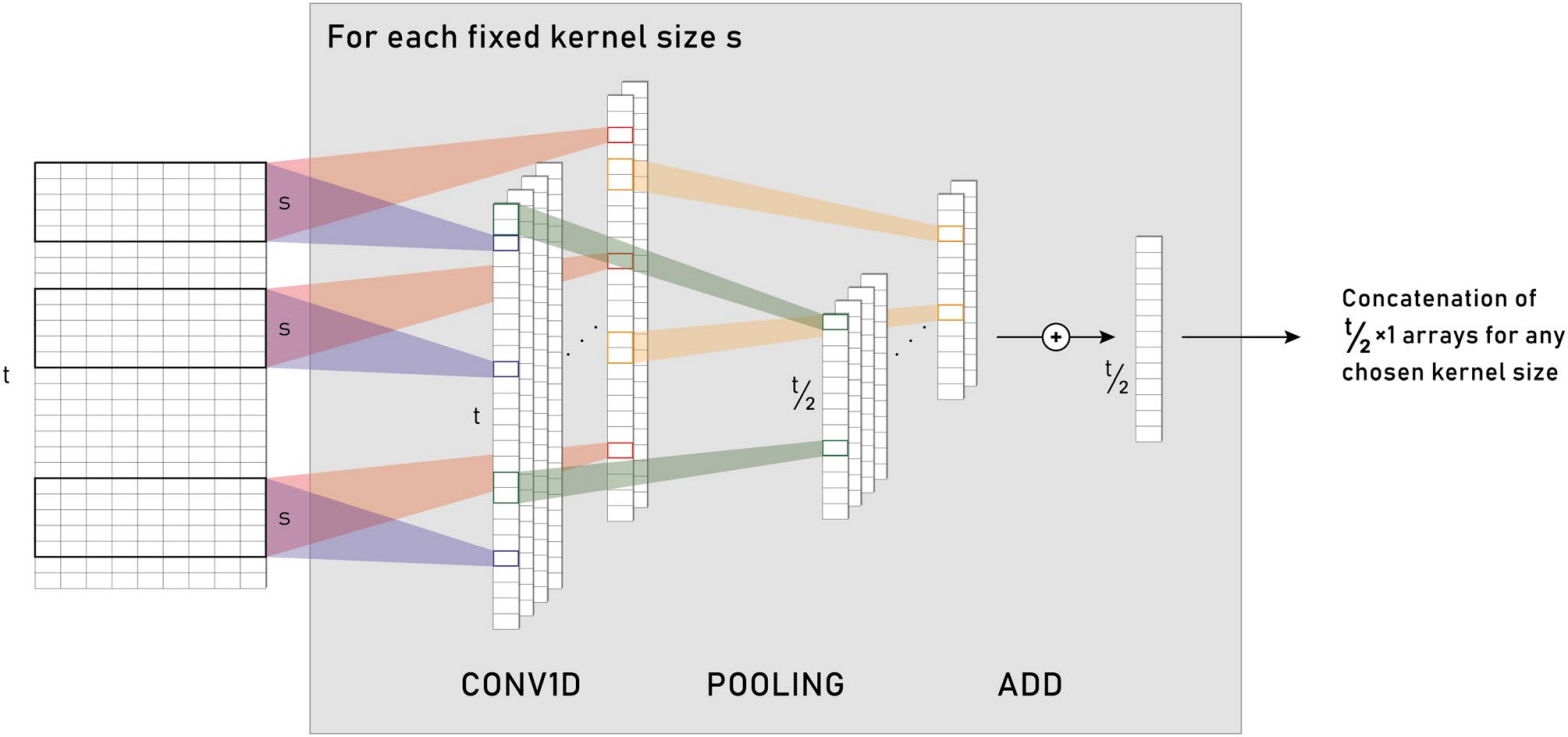
A representation of the TCNN block. From the left side: for a fixed kernel size *s*, starting from a temporal matrix with $t$ time steps and $d$ features, 64 vectors are generated using $s \times d$*filters* through an 1D convolution; zero padding is chosen to obtain vectors of the same dimensions, that is $t \times 1$. Different colors represent the different filters where the convolution appears for clarification. After the convolution operation, the filtered arrays are passed through a *pooling layer*, where the size of the pooling is set to 2 and therefore the dimension of the pooled filtered arrays is halved to $\frac{t}{2} \times 1$. Experimental tests have confirmed that the choice of both the maximum and average pooling improves the model accuracy. To reduce the dimension of the forthcoming concatenation, an *addition layer* is inserted, which returns a single vector with size $\frac{t}{2} \times 1$. The concatenation at the right corresponds to the concatenation of all the output vectors obtained for the different choices of the kernel size *s*.

The design of our proposed method has been done to explore and extrapolate information from the temporal evolution of the medical booking history of the patients; a previous work, done by Cheng et al., proposed *Temporal Fusion Convolutional Neural Networks (TFCNN)*^8^ study the clinical history as a matrix. These Neural Networks encode the input matrices into smaller but more meaningful features with a concatenation of *temporal discrete convolution* and *pooling* operations; then, such reduced features are passed to a fully connected Neural Network, to non-linearly combine them. Such networks have been applied with good results to chronic obstructive pulmonary disease (COPD) risk prediction^18^, where, similarly to this case, the input is composed of the clinical history and other features that are characteristic of the patient. Due to the particular characteristics of our data set, Temporal Convolutional Neural Networks have to be used. The difference between the aforementioned work and this work is that our data set does not contain any clinical history, but only bookings to provisions, therefore a careful adjustment to the model must be done. The result of this operation is a feedforward neural network model composed of a structured temporal convolutional layer and a multilayer perceptron. In detail, the Temporal CNN (TCNN) block takes as input a $t \times d$ matrix ($t=52$ and $d=8,19$ in our application) and a set *S* of $n_S$ sizes as parameters ($S=\{6,9,\dots ,51\}$ in experimental tests). For each $s \in S$, 64 filters with a $s \times d$ kernel operate on the input matrix to discover different patterns, obtaining 64 $t \times 1$ arrays to which a $2 \times 1$ pooling is applied. At this point the $\frac{t}{2} \times 1$ are added to produce a single vector. The $n_S$ vectors obtained by varying $s \in S$ are then concatenated. Hence, the output of the TCNN block is an $n_S \cdot \frac{t}{2}$ column vector. Figure 3 summarizes the above process. The convolutional layer of the proposed method is composed of four TCNN blocks, to apply a maximum or an average pooling to each temporal matrix. Next, by concatenating the output column vectors also with the gender and age features, the vector of $4 \cdot n_S \cdot \frac{t}{2} + 2$ components becomes the input of a feedforward fully connected neural network with two hidden layers (respectively of 128 and 32 perceptrons) and an output layer composed of 11 units, which generates the final output of the method. Figure 4 shows the pipeline described.

### Evaluation metrics

Given that this study focuses on a multi-label classification problem, the evaluation of the models will take into account two types: by using a cumulative metric for all the labels, or by applying a single metric for each label. In the former methodology, three metrics were used for the comparison: the *Hamming Distance*, the *Exact Accuracy* and the *Top 10 Facilities Exact Accuracy*, while for the latter methodology, several metrics (accuracy, precision, recall, F1-score, and AUC) were estimated for each label. Hamming distance $d_{H}$ represents the average number of missed labels for each window, i.e. by the following formula:

$$\begin{aligned} d_{H}(y,{\hat{y}}) = \Big | \left\{ \, j \, | \, y_j \ne {\hat{y}}_j \right\} \Big | \end{aligned}$$

where |*S*| is the cardinality of the set *S*, ${\hat{y}} \in \{0,1\}^{11}$ is the predicted output of the model.

Meanwhile, Exact Accuracy represents the percentage of the correctly predicted samples:

$$\begin{aligned} \text {Exact Accuracy} = \frac{\text {Exact Predicted Samples}}{\text {Total Test Samples}} \end{aligned}$$

where an exact prediction occurs if the vector of the predicted label ${\hat{y}}$ is equal to the vector of the true label *y*.

Finally, Top 10 Facilities Exact Accuracy is defined as the Exact Accuracy of the predicted output when the last label is ignored, since it is just a workaround for the implementation of the BP-MLL algorithm for our proposed model.

For a fixed label, from the confusion matrix:

**Table:** 

Prediction	Actual	
	0	1
0	VN	FN
1	FP	VP

it is straightforward to calculate:

$$\begin{aligned} \text {accuracy}= & {} \frac{\text {TP}+\text {TN}}{\text {TP}+\text {FP}+\text {TN}+\text {FN}} ,~~ \text {precision} = \frac{\text {TP}}{\text {TP}+\text {FP}} ,~~ \text {recall} = \frac{\text {TP}}{\text {TP}+\text {FN}} , \\ \text {F1-score}= & {} \frac{2 \times \text {precision} \times \text {recall}}{\text {precision}+\text {recall}} \end{aligned}$$

where $\hbox {TP}$ is the true positive, $\hbox {FP}$ is the false positive and $\hbox {FN}$ is the false negative. The AUC score is defined as the area under the ROC curve, used to measure how well the model can distinguish between two classes, even more so when the labels are unbalanced.

## Results

In this section, our experimental results are shown. Firstly, the whole data set was considered, but only 37.5% of windows had at least one of the first ten labels equal to 1, i.e. at least one appointment in any of the facilities which we focused on. Therefore, before each run, we randomly reduced the remaining 62.5% of windows to a seventh of their number to increase the percentage of the positive cases for each label. Table 7 shows how the frequencies changed.

**Table 7:** The frequencies of the medical facilities before and after the removal of six seventh of the samples which have only label 11 set to 1.

Medical facility	Frequencies (before)	Frequencies (after)
1	0.056	0.120
2	0.050	0.107
3	0.049	0.106
4	0.044	0.094
5	0.042	0.091
6	0.042	0.090
7	0.041	0.087
8	0.039	0.082
9	0.037	0.079
10	0.035	0.075
11	0.728	0.415

The associated medical facilities are referred from Table 3.

In each run, this new data set was split into a training set (90%) and test set (10%) for RF, while it was split into a training set (72%), validation set (18%), and test set (10%) in the case of MLP and our proposed method. Regarding this different rule of splitting, we clarify that the validation set is only used in the early stopping criteria related to the Adam parameter optimization algorithm^19^. In fact, due to long execution times, we did not apply any kind of semi-automatic search algorithm; in a previous testing phase, the hyperparameter tuning of each model was performed by analyzing the results of several test runs. After the testing phase, we evaluated the considered methods by performing 15 different runs for each of them, differing for the simple randomization of the samples in the data set before each splitting.

Tables 8 and 9 show that the proposed method outperforms the traditional method according to any of the considered metrics. Each listed measurement is in the form *mean* ± *standard deviation*, calculated from the results of all the runs. RF and MLP miss on average one every 14 labels, while the proposed method misses on average less than one every 26 labels ($11{/}0.42087\approx 26.14$).

**Table 8:** Starting from the results of 15 different runs, the table shows the results of certain metrics in the form *mean* ± *standard deviation* for the considered methods.

Method	Hamming distance	Exact accuracy	Top 10 facilities exact accuracy
RF	$0.82063\pm 0.00303$	$0.48430\pm 0.00164$	$0.59654\pm 0.00130$
MLP	$0.80802\pm 0.00125$	$0.48745\pm 0.00131$	$0.60261\pm 0.00104$
Proposed	**0.42087 ± 0.01977**	**0.65509 ± 0.01465**	**0.76854 ± 0.01101**

The best scores are highlighted in bold. It is noticeable that the proposed method outperformed the others according to any of the metrics used.

**Table 9:** Starting from the results of 15 different runs, the table shows some metrics in the form *mean* ± *standard deviation* for the methods considered.

Method	Overall precision	Overall recall	Overall F1-score	Overall AUC
RF	$0.77621\pm 0.00189$	$0.52943\pm 0.00073$	$0.61249\pm 0.00010$	$0.91161\pm 0.00049$
MLP	$0.73660\pm 0.00161$	$0.58937\pm 0.00343$	$0.64605\pm 0.00186$	$0.91200\pm 0.00122$
Proposed	$0.82082\pm 0.00945$	$0.89809\pm 0.00419$	$0.85733\pm 0.00644$	$0.96181\pm 0.00140$

The proposed method outperformed the others, in particular with respect to the overall recall, which means that it produced far fewer false positives that RF and MLP.

In particular, the Overall Recall referring to our method (Table 9) reveals that even though the label frequencies are very low, the proposed model is capable to recognize the positives with a better accuracy than the other tested methods. Further investigations were carried out for each facility. Table 10 shows the accuracy of our method referring to each facility; it can be observed that the model produces a very high accuracy for every label.

**Table 10:** Accuracy of the proposed model for each facility.

Facility	1	2	3	4	5
Accuracy	$0.977\pm 0.001$	$0.961\pm 0.003$	$0.962\pm 0.002$	$0.981\pm 0.001$	$0.978\pm 0.001$

Facility	6	7	8	9	10
Accuracy	$0.973\pm 0.001$	$0.972\pm 0.001$	$0.973\pm 0.002$	$0.981\pm 0.001$	$0.971\pm 0.002$

The mean accuracy on the test set is over 97%, a further measure of the validity of model. Even if it is out of our focus, we also verified the accuracy on the 11th label and this proved to be similarly high (over 85%).

Once we had gathered evidence about the quality of the proposed model, we investigated cases of patients who had not had any appointment in a particular facility *j* in the 52 weeks but also had had an appointment in the same facility *j* in the following 9 weeks. In particular, the aim was to understand the capability of the proposed model to predict the first access of patients to a facility. Table 11 shows the accuracy with reference to each facility; the mean accuracy on this specific set of patients is over 64% ($\pm 0.0156\%$).

**Table 11:** The table shows how the proposed method predicts the number of accesses of patients to a facility where they have never previously been; the overall accuracy is over 64%.

Facility	1	2	3	4	5
Accuracy on 1st access	$0.695\pm 0.012$	$0.682\pm 0.014$	$0.687\pm 0.011$	$0.603\pm 0.044$	$0.684\pm 0.020$

Facility	6	7	8	9	10
Accuracy on 1st access	$0.697\pm 0.017$	$0.637\pm 0.022$	$0.609\pm 0.026$	$0.525\pm 0.033$	$0.643\pm 0.021$

**Figure 5:**
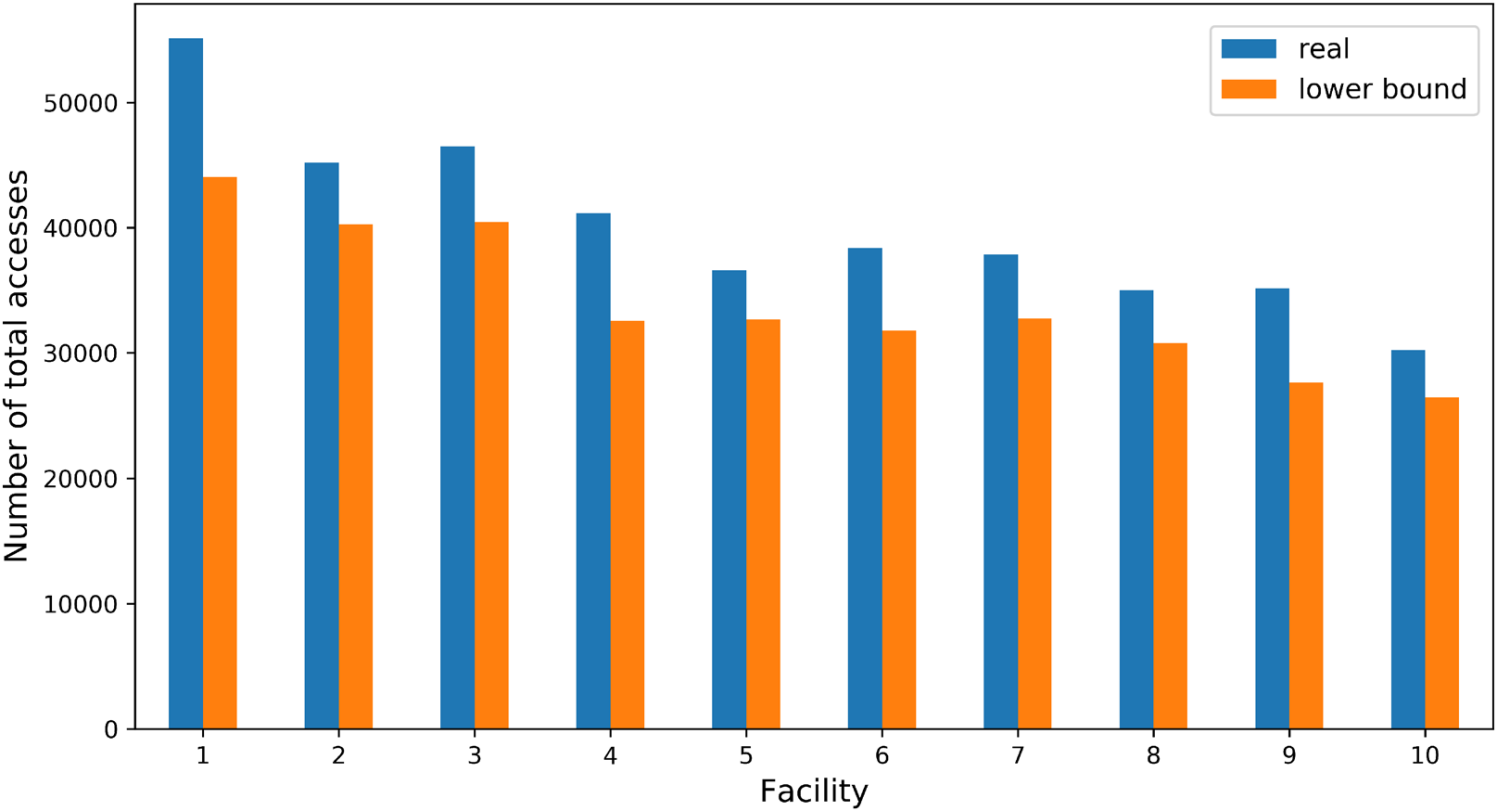
The histogram summarizes the total number of accesses and the number of predicted positives obtained from a single run of the proposed model. As in the average of all the runs, the number of the predicted positives on average is almost 85% of the real number.

As described above, starting from the previous year’s clinical history the proposed method attempts to predict if and in which facility a patient will have at least one appointment. By this feature, this method may be useful to the health authority, because it can provide a non-trivial lower bound on the facility patient attendance in terms of patient access. Such comparison has been done by running the proposed model 15 times in the same way as in the results with the other models, and then we compared the total of predicted positives with the real number of accesses to facility present in such test set. This has been done to keep the coherency of the statistical analysis. Figure 5 illustrates a single run of the comparison, but if we consider the average and the standard deviation of all the runs, we obtained that the difference between the lower limit and the real amount is $16 \pm 4\%$ of the latter.

## Conclusions

Healthcare technologies are not necessarily confined to patient management and care but can also have a great significance concerning prevention. The greater is the emphasis placed on preventive medicine rather than the mere treatment of the symptoms, the more effective will the healthcare system be for everyone, both for the healthcare management and the wider community. In this paper we have presented a Temporal Fusion CNN adapted for the multi-label problem described. Starting from only administrative information about the previous year’s clinical history of a patient, the goal of the model has been to predict if and in which facility the patient will book an appointment. Even though the administrative data do not store information about medical examination outcomes or reports, the experimental results prove the quality of the model constructed. As future research, with additional information about patients and HS provisions, it would be interesting to evaluate the model predictions for patient health, not only from that of administration. In the latter part of the work, we have proposed a patient attendance lower bound for each facility as a service to the health authority. As a further future research project, supported by supplementary financial information, it may also be possible to provide statistical data to optimize the funding distribution of the regional health system.

## Hardware and software

The hardware used in this study consists of a desktop computer with an 8-core Intel Core i9-9900K CPU, together with a GPU NVidia RTX 2080 Ti, all mounted on an Asus PRIME Z390-A motherboard with 128 GB of RAM and 2 TB of SSD. The GPU has been used to speed up the training of the deep learning models. The programming language adopted is Python version 3.7.6, with the libraries Numpy 1.18.1 and Pandas 1.0.1 for the data set, scikit-learn 0.22.1^20^ for the implementation of RF and Tensorflow 2.0^21^ with GPU support for the deep learning models.
